# Cardiac Mass: A Case of Cardiac Papillary Fibroelastoma

**DOI:** 10.7759/cureus.3482

**Published:** 2018-10-23

**Authors:** Roshni Jain, Lohitha Kolli, Shravani R Vindhyal, Shilpa Kshatriya, Mohinder R Vindhyal

**Affiliations:** 1 Internal Medicine, University of Kansas School of Medicine-Wichita, Wichita, USA; 2 Radiology, University of Kansas School of Medicine-Wichita, Wichita, USA; 3 Cardiology, University of Kansas School of Medicine-Wichita, Wichita, USA

**Keywords:** cardiac imaging, cardiac pathology, cardiac surgery, cardiac mass

## Abstract

Cardiac papillary fibroelastoma (CPF) is one of the most common neoplasms of the cardiac valvular structures that are associated with complications such as systemic stroke, embolism, and arrhythmias. We present a case of an incidentally discovered left ventricular mass in a 75-year-old Caucasian woman.

## Introduction

Primary cardiac tumors occur infrequently. Approximately 80% of those that do occur are benign and the remaining 20% are malignant [1]. Cardiac papillary fibroelastomas (CPF) are the second most common benign cardiac tumors but are six times less likely to occur than atrial myxomas, the most common benign cardiac tumor [2]. Although the exact prevalence of CPF remains unknown, the incidence of CPF, often diagnosed due to the incidental findings on echocardiography, is increasing as evidenced by the mounting number of case reports in the literature [1]. CPF is a rare and benign tumor, which can be seen anywhere in the heart but usually involves cardiac valves such as an aortic valve. Here we report the case of an incidentally discovered left ventricular papillary fibroelastoma detected by echocardiography in a 75-year-old Caucasian woman with successful surgical resection.

## Case presentation

**Figure 1:**
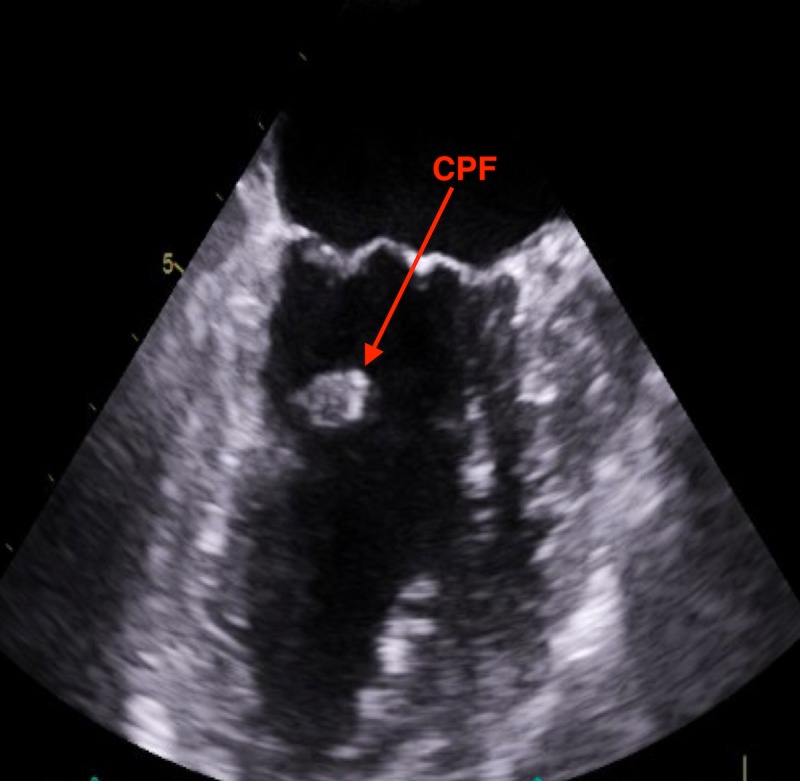
TEE showing mass in the subchordal apparatus of the anterior mitral leaflet TEE: transthoracic echocardiogram

**Figure 2:**
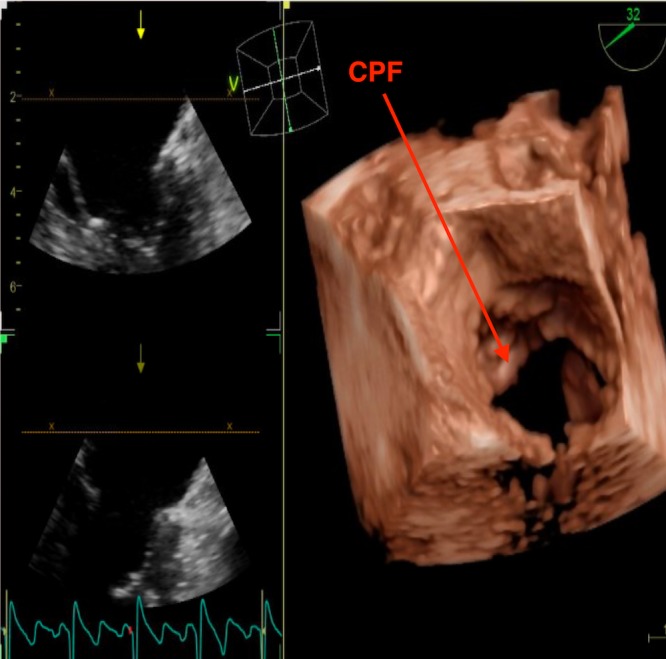
Three-dimensional EKG showing the same subchordal mass on the anterior leaflet of the mitral valve EKG: echocardiogram

A 75-year-old Caucasian woman with a past medical history significant for hypertension, dyslipidemia, and depression was admitted to our hospital for evaluation of an incidentally discovered left ventricular mass. The patient denied having a history of stroke, peripheral embolization, or myocardial infarction. The patient reported compliance with her medications and had been taking furosemide 20 mg, amlodipine/benazeprilat 5/20 mg, rosuvastatin 40 mg, aspirin 81 mg, sertraline 100 mg, zolpidem 5 mg, and omega-3 fatty acids/fish oil. The patient reported food allergies to bananas, pecans, and walnuts. The patient denied any significant surgical history. Her social history was significant for tobacco abuse in the past but denies current use of tobacco products, alcohol, and recreational drugs. Transthoracic echocardiography (TTE) was performed for shortness of air and revealed the left ventricular mass. The patient then underwent a transesophageal echocardiogram (TEE) for further evaluation, which localized the mass to the subchordal apparatus of the anterior mitral valve leaflet (Figures 1-2).

**Figure 3:**
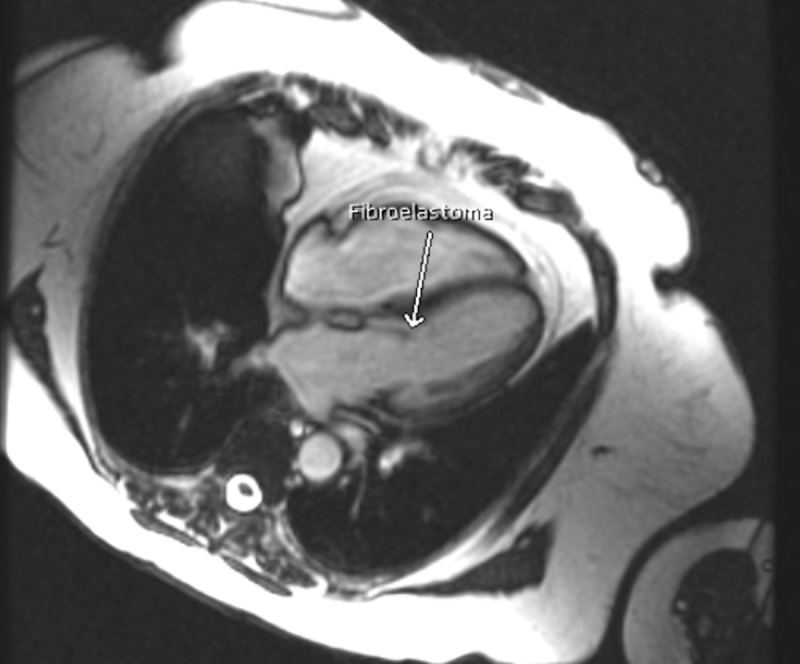
Coronal section of the heart: cardiac MRI (CMR) showing fibroelastoma attached to the subchordal apparatus of the anterior mitral valve leaflet MRI: magnetic resonance imaging; CMR: cardiac magnetic resonance imaging

The mass was globular and non-pedunculated and measured 1.9 cm within the chordae tendinae. The echo dense core differentiated the globular mass from vegetation or thrombus. The rest of the echocardiography findings showed normal left ventricular systolic function with a normal ejection fraction of 55% to 60%. The patient was then referred to cardiothoracic surgery, who wanted cardiac magnetic resonance imaging (CMR) for better identification and visualization of the mass. CMR revealed a 1.8 x 1.0 x 0.3-cm lesion on the anterior leaflet of the mitral valve (Figure 3).

Consent was obtained, and the patient agreed to resection to decrease the risk of stroke, myocardial infarction, peripheral embolization, and sudden death. Prior to the surgical resection, the patient underwent coronary angiography to look for patency of the coronary vessels, which demonstrated a left dominant arterial supply system with no significant obstructive coronary artery disease and TIMI-3 flow in all the coronary vessels. Left ventricular end-diastolic pressure (LVEDP) was mildly elevated at 19 mm Hg, indicative of mild diastolic dysfunction. Preoperative electrocardiogram (EKG) was normal and showed that the patient was in normal sinus rhythm. During the surgery, gentle retraction of the aortic valve leaflets allowed visualization of the mass.

**Figure 4:**
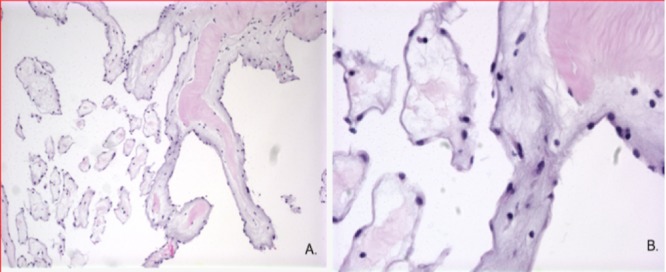
Fibroelastoma H&E 10x (A) and 40x (B): narrow elongated branching papillary fronds composed of central avascular collagen and variable elastic tissue surrounded by acid mucopolysaccharide and lined by hyperplastic endothelial cells H&E: hematoxylin and eosin

The mass was located within the left ventricle attached to the papillary muscle and chordae tendinae of the anterior leaflet of the mitral valve. The mass was shaved off without difficulty. The tumor was flushed in appearance, rubbery to spongy in texture, and 1.7 x 1.1 x 0.4 cm in size. No residual tumor was left behind. The tumor was thought to be a myxoma upon macroscopic inspection. No complications were encountered during the surgery. Histological examination of the mass from the pathology revealed narrow, elongated, and branching papillary fronds composed of central avascular collagen and variable elastic tissues surrounded by acid mucopolysaccharide and lined by hyperplastic endothelial cells (Figure 4).

**Figure 5:**
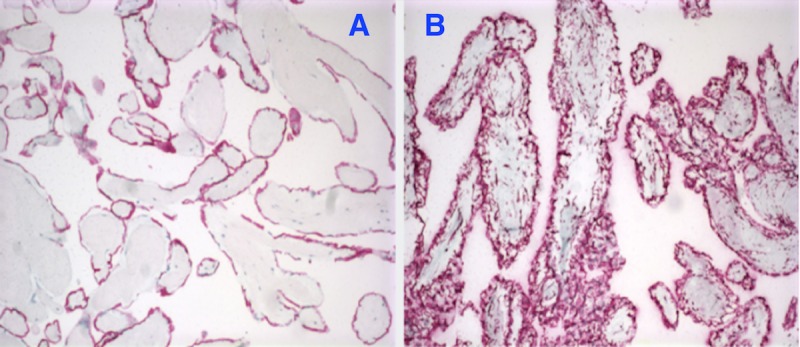
Left (A): surface lining showing cells staining positive for CD-34; Right (B): surface lining showing intermediate cells staining positive for vimentin

The surface lining cells were highlighted with CD-34, and the intermediate cells stained with vimentin and S-100 (Figure 5). These findings confirmed the diagnosis of CPF. 

Immediately post-op, the patient was confused, light-headed, and complained of dyspnea on exertion. These problems resolved by post-op day two. Following this, the patient’s post-operative course was uneventful. She was discharged to home on post-op day six with home health care.

## Discussion

CPFs are the second most common benign cardiac tumors but are still six times less likely to occur than atrial myxomas, which occur most frequently [2]. Primary cardiac tumors are rare entities with the incidence estimated between 0.001% and 0.3% [3]. Of these, approximately 71% are benign and the remaining 29% are malignant [4]. CPFs have been documented in patients ranging from 11 months to 86 years of age but mostly occur in patients 55 and older and is more likely to occur in women than men [5-6]. Ninety percent of the CPFs reported were located on valves, primarily the aortic valve (44.5%) and the rest on the mitral leaflets (36.4%); only eight were located on the left ventricular surface with the remaining thirty-two were located on the atrial surface [4]. Twenty-three of the 40 were localized to the anterior leaflet and 17 to the posterior leaflet [5]. The CPF is presumed to be a reactive process rather than a true neoplasm [7]. It is postulated that CPF arises secondary to small thrombi coalescing on valves at the site of endothelial damage. The tumor is thought to grow in size as microthrombi accumulate on the initial nidus [2]. CPFs are found most commonly as incidental findings on EKG where the tumors exhibit characteristic findings. Typically, the mass may be round, oval, or irregular in shape with a homogenous texture and well-demarcated borders. Ninety-nine percent of masses are less than 20 mm in the largest dimension, and roughly half of the lesions may rest on mobile stalks [5]. Stippling around the border of the tumor and speckling throughout the mass might also be appreciated. Macroscopically, CPFs have been described as resembling cauliflower, fronds, and sea anemones [8]. Microscopically, the tumors are composed of an endothelium-encased acid mucopolysaccharide layer and an inner avascular core consisting of collagen, smooth muscle cells, and elastic fibers [5].

Although CPFs themselves are benign, serious complications may arise from embolization of the tumor, leading to stroke, transient ischemic attack, acute myocardial infarction, angina pectoris, ventricular arrhythmia, and even sudden death [2]. Such complications are uncommon but are more likely to occur if the mass is on the left side of the heart, as it was in our patient. Surgical resection of the tumor is the only method by which one can completely eliminate the risk of embolization. If the patient is experiencing symptoms secondary to the tumor, then surgery is recommended. If the patient is asymptomatic, then the approach is less clear. Twenty-two percent of the patients with CPF who were not surgically treated suffered from neurological embolization complications, and four percent suffered peripheral complications [8]. Once the tumor is removed completely, the chance of recurrence appears to be low, and there are no compelling data to continue anticoagulation in the long term unless other indications to do so are present [9].

## Conclusions

Thus far, studies have been done to characterize CPF. The next step is a retrospective study in which the risk factors for cardiovascular diseases, such as hypertension and dyslipidemia as seen in our patient, are assessed against the presence of CPF. Furthermore, longitudinal follow-up studies are needed to determine the rate of recurrence of CPF following surgical resection.
